# Lyngbyastatins 8–10, Elastase Inhibitors with Cyclic Depsipeptide Scaffolds Isolated from the Marine Cyanobacterium *Lyngbya semiplena*

**DOI:** 10.3390/md7040528

**Published:** 2009-11-03

**Authors:** Jason C. Kwan, Kanchan Taori, Valerie J. Paul, Hendrik Luesch

**Affiliations:** 1 Department of Medicinal Chemistry, University of Florida, 1600 SW Archer Road, Gainesville, FL 32610; USA; E-Mails: jkwan@ufl.edu (J.C.K.); ktaori@ufl.edu (K.T.); 2 Smithsonian Marine Station, 701 Seaway Drive, Fort Pierce, FL 34949, USA; E-Mail: paul@si.edu (V.J.P.)

**Keywords:** lyngbyastatins, cyanobacteria, Lyngbya semiplena, cyclic depsipeptides, elastase inhibitors

## Abstract

Investigation of an extract from the marine cyanobacterium *Lyngbya semiplena*, collected in Tumon Bay, Guam, led to the identification of three new cyclodepsipeptides, lyngbyastatins 8–10 (**1**–**3**). The structures of **1**–**3** were determined by NMR, MS, ESIMS fragmentation and chemical degradation. Compounds **1**–**3** are closely related to lyngbyastatins 4–7. Like the latter compounds, we found **1**–**3** to inhibit porcine pancreatic elastase, with IC_50_ values of 123 nM, 210 nM and 120 nM, respectively.

## 1. Introduction

**Figure 1.**
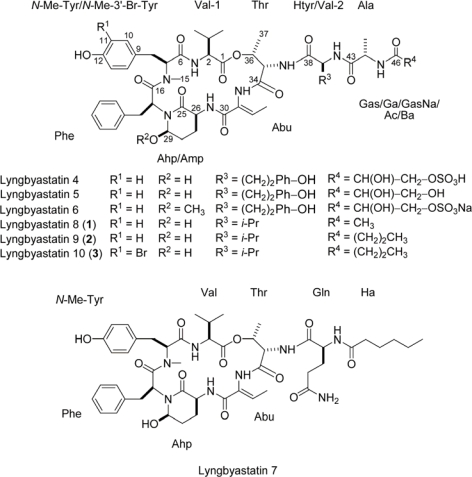
Structures of lyngbyastatins 4–7 and compounds **1**–**3**.

Marine cyanobacteria have yielded many novel and bioactive secondary metabolites [1], including many cytotoxins [2–4]. Our efforts have been focused on discovering novel cyanobacterial metabolites with cytotoxic and other activities. Indeed, we have recently identified several cyanobacterial compounds that are potent protease inhibitors, such as grassystatins A–C that selectively inhibit cathepsin E [5] and lyngbyastatins 4–7 [6,7] (see Figure 1) which inhibit porcine pancreatic elastase. The latter group are part of a class prolifically produced by marine and aquatic cyanobacteria [8–10], containing 3-amino-6-hydroxy-2-piperidone (Ahp) as part of a six-unit cyclic core with a pendant side chain. These generally inhibit certain serine proteases, due to extensive complementarity to the enzymes’ active sites. Additionally, the cyclic core is very rigid, due to hydrogen bonds from the Ahp unit to the opposite Val, making hydrolysis of the inhibitor difficult for the protease enzymes [11–13]. Elastase is particularly susceptible to the lyngbyastatin series of this inhibitor prototype, which contain 2-amino-2-butenoic acid (Abu) adjacent to the Ahp unit [6,7,14]. In humans, neutrophil elastase is known to contribute to immunogenic tissue damage in diseases such as cystic fibrosis and asthma [15]. We now report three new members of this class, lyngbyastatins 8–10 (**1**–**3**, see Figure 1), which are close relatives of lyngbyastatins 4–7.

## 2. Results and Discussion

A sample of *Lyngbya semiplena* was collected in Tumon Bay, Guam. Samples were freeze-dried and stored frozen for ~8 years before extraction with EtOAc–MeOH (1:1). The resulting non-polar extract was partitioned between hexanes and MeOH–H_2_O (80:20). The MeOH–H_2_O fraction was further partitioned between *n*-BuOH and H_2_O. The *n*-BuOH fraction was subjected to silica and reversed-phase chromatography and finally HPLC to furnish **1** (1.8 mg), **2** (1.1 mg) and **3** (~100 μg).

HRESI/APCIMS and NMR data for **1** suggested a molecular formula of C_47_H_64_N_8_O_12_ (*m/z* 955.4524 for [M + Na]^+^, and 915.4599 for [M + H – H_2_O]^+^). The ^1^H-NMR spectrum suggested that **1** was a depsipeptide, with several exchangeable amide proton signals (~δ_H_ 6–8) and α-protons (~δ_H_ 4–5.5). The most downfield α-proton also had a corresponding downfield ^13^C shift (δ_H_/δ_C_ 5.53/71.4), as shown in the edited HSQC spectrum (see Table 1), indicating a methine adjacent to an ester linkage. In addition, one *N*-methyl was present (δ_H_ 2.74). A methyl singlet at δ_H_ 1.82 suggested the presence of an acetyl group, and there was a downfield singlet (δ_H_ 9.38) characteristic of a phenolic OH in DMSO. The ^1^H-NMR spectrum of **1** was strikingly similar to the spectra of lyngbyastatins 4–7 [6,7], and indeed examination of the COSY, edited HSQC, HMBC, ROESY and TOCSY spectra of **1** revealed the same units present in the cyclic core as some other members of the series: Thr, Val, *N*-Me-Tyr, Phe, Ahp and Abu. Like previously described lyngbyastatins, there was a general paucity of HMBC signals within the cyclic core, but examination of ROESY correlations made it apparent that **1** had the same cyclic core as lyngbyastatins 4–7. Unlike the latter, **1** contains only hydrophobic residues in the pendant side chain, with Val replacing Htyr and an acetyl group replacing Gas/Ga/GasNa (Table 1 and Table S1, Supporting Information).

NMR data for **2** suggested a close analog of **1**. HRESI/APCIMS data suggested a molecular formula of C_49_H_68_N_8_O_12_ (*m/z* 983.4823 for [M + Na]^+^, and 943.4898 for [M + H – H_2_O]^+^), a difference of C_2_H_4_ from **1**. The acetyl methyl singlet (δ_H_ 1.82) observed in **1** is absent in **2**, suggesting that this unit is extended in the latter. Examination of the ^1^H-NMR, COSY, edited HSQC, HMBC, ROESY and TOCSY of **2** in DMSO-*d*_6_ (see Table 1 and Table S2, Supporting Information) revealed the presence of the same units as for **1**. However, in place of acetyl, a butanoic acid unit (Ba) was deduced. The HMBC and ROESY correlations were very similar to those observed for **1**. Along with the close similarity in chemical shifts, NMR data allowed the assembly of the structure shown (Figure 1).

Compound **3** was obtained in much lower yield (~100 μg). The HRESI/APCIMS spectrum contained ion clusters characteristic of species containing one bromine atom, with two major peaks 2 amu apart in a ratio close to 1:1, and the molecular formula was calculated to be C_49_H_67_N_8_O_12_Br (*m/z* 1061.3951 and 1063.3944 for [M + Na]^+^, 1021.4028 and 1023.4033 for [M + H – H_2_O]^+^). This is the same molecular formula as **2**, except one Br atom was present in place of a hydrogen atom. Additionally, proton and carbon chemical shifts were very similar to **2**. Examination of the ^1^H-NMR, COSY, edited HSQC and ROESY spectra of **3** in DMSO-*d*_6_ (see Table 1 and Table S3, Supporting Information) suggested the presence of the same units as in **2**, except that there were only signals for three aromatic protons attributable to the *N*-Me-Tyr unit, including a singlet (δ_H_ 7.25), indicating that either *N*-methylated 2’- or 3’-Br-Tyr was present. Chemical shift data and ROESY correlations between the Br-Tyr singlet to both β-methylene protons (see Table 1 and Table S3, Supporting Information), supported the proposal that the bromine atom is present at the 3’ position. This is consistent with previously reported compounds in this series such as kempopeptin B [14] and cyanopeptolin 954 [16], which possess bromine and chlorine at the 3’-position of Tyr, respectively.

**Figure 2.**
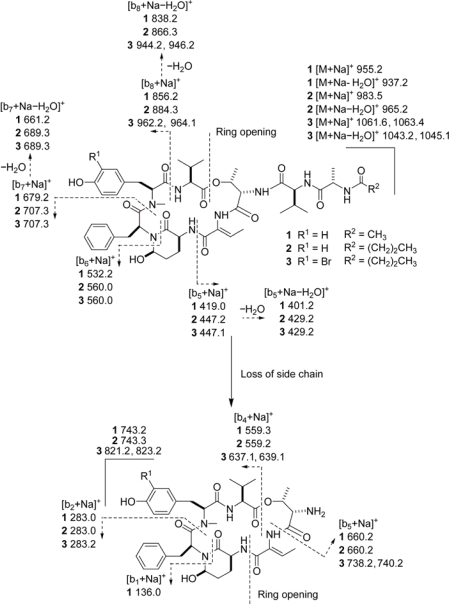
ESIMS fragmentation pattern for lyngbyastatins 8–10 (**1**–**3**). Two series of b ions were observed. In one (top), ring opening at the ester bond occurred, followed by sequential loss of units. In the other (bottom), the side chain was lost, before ring opening at the amide bond between Ahp and Abu. For comparison, see ESIMS fragmentation pattern of lyngbyastatin 7, Figure S1. Note: All ions retained Na^+^.

Further evidence for the assigned 2D structures of **1**–**3** was obtained by ESIMS fragmentation (Figure 2). Notably, this data unambiguously placed the Br atom in **3** with the *N*-Me-Tyr unit. Fragmentation data for a related series of cyclic peptides, the aeruginopeptins, has been reported [17]. We found a similar fragmentation pattern using an authentic sample of lyngbyastatin 7 [7], plus another series that was consistent with loss of the pendant side chain followed by ring cleavage between Ahp and Abu (Figure S1, Supporting Information).

The configuration of the Abu unit was established for **1** by a ROESY correlation between H_3_-33 and the Abu NH (see Table S1, Supporting Information), indicating *Z* configuration. In **2**, this correlation was not observed. However, an unusual 4-bond HMBC correlation was seen between C-30 and H_3_-33 (see Table S2, Supporting Information). Such 4-bond correlations are typically observed in substructures where the bonds between the H and C atoms can form a “w” configuration [18–20]. This correlation therefore supports *Z* configuration for Abu in **2**. Configuration of Abu in **3** could not be determined by ROESY, but it is presumed to be *Z* on the basis of proton chemical shifts in this unit, which are very close to those in **1** and **2**, and on biogenetic grounds. If the configuration were *E*, it is likely that these shifts would be different due to the anisotropic influence of the nearby carbonyl group (C-30). The absolute configurations of other units in **1**–**3** were determined by FDLA-based Marfey’s analysis [21], using both UV and MS detection, to all be the l-form. The configuration of C-3 of the Ahp unit was determined by CrO_3_ oxidation followed by hydrolysis to liberate l-Glu, as described previously [6,7]. The configuration of C-6 of Ahp was determined by examination of ROESY correlations within the unit and by considering coupling constants, such that (3*S*,6*R*) configuration was assigned for this unit. CrO_3_ oxidation was not carried out for **3** due to lack of material, but the Ahp unit is most likely the same configuration as in **2** and **3**, based on overall similarity of NMR data and the fact that the result of Marfey’s analysis was the same for other units.

Compounds **1**–**3** were assessed for inhibition of porcine pancreatic elastase. Lyngbyastatin 7, tested concurrently, exhibited an IC_50_ of 47.3 ± 7.6 nM in this assay. By comparison, **1**–**3** were all less potent, with IC_50_ values of 123 ± 2 nM, 210 ± 10 nM, and 120 ± 16 nM, respectively. Compounds **1**–**3** possess the same depsipeptide core as lyngbyastatin 7. And therefore, differences in the side chain residue may play some part in their reduced potency. The presence of exclusively hydrophobic residues in the pendant chain may allow for fewer favorable electrostatic interactions and hydrogen bonding with the enzyme. Conversely, the presence of Br does not seem to have much of an influence of the potency of **3** compared with **1** and **2**.

## 3. Experimental Section

### 3.1. General Experimental Procedures

Optical rotation was measured on a Perkin-Elmer 341 polarimeter. UV spectra were obtained on a SpectraMax M5 (Molecular Devices) and IR data were obtained on a Bruker Vector 22 instrument. ^1^H and 2D NMR spectra for **1**–**3** in DMSO-*d*_6_ were recorded on a Bruker 600 MHz Avance II Spectrometer, using a 1-mm triple-resonance high-temperature superconducting cryogenic probe [22] for **1** and **2**, and a 5-mm cryogenic probe for indirect detection (Bruker CryoProbe TXI) for **3**. Spectra were referenced to residual solvent signals [δ_H/C_ 2.49/39.5]. HSQC experiments were optimized for ^1^*J*_CH_ = 145 Hz, and HMBC experiments were optimized for *^n^J*_CH_ = 7 Hz. HRESI/APCIMS data were recorded on an Agilent LC-TOF mass spectrometer equipped with an APCI/ESI multimode ion source detector in positive ion mode. LC-MS data were obtained using an API 3200 triple quadrupole MS (Applied Biosystems) equipped with a Shimadzu LC system. ESIMS fragmentation data were obtained on an API 3200 by direct injection with a syringe driver. Enzymatic assays were read using a SpectraMax M5. The standard for *N*-Me-3’-Br-Tyr was prepared as previously described [23].

### 3.2. Extraction and Isolation

A sample of *Lyngbya semiplena* was collected from Tumon Bay, Guam on December 17, 1998. The freeze-dried organism (dry weight 1.85 kg) was extracted with EtOAc–MeOH (1:1). The extract was concentrated to dryness and partitioned between hexanes and MeOH–H_2_O (80:20). After removal of the solvents, the latter fraction was further partitioned between *n*-BuOH and H_2_O. The *n*-BuOH soluble fraction was subjected to silica gel chromatography, using a gradient system of increasing *i*-PrOH in CH_2_Cl_2_. The fraction eluting with 50% *i*-PrOH was further purified by reversed-phase chromatography, using a gradient system of increasing MeOH in H_2_O. The fraction eluting with 80% MeOH was purified by reversed-phase HPLC (YMC-Pack ODS-AQ, 250 × 10 mm, 2.0 mL/min; UV detection at 220 and 254 nm) using a MeOH–H_2_O linear gradient (20–100% over 60 min, then 100% MeOH for 10 min), to furnish compounds **1** (*t*_R_ 42.8 min; 1.8 mg), **2** (*t*_R_ 47.0 min; 1.1 mg) and impure **3** (*t*_R_ 49.7 min). Compound **3** was repurified using a different column (Phenomenex Synergi Hydro-RP, 250 × 10 mm, 2.0 mL/min, PDA detection, 200–800 nm), with the same linear gradient used before to yield pure **3** (*t*_R_ 47.7 min; ~100 μg).

### 3.3. Lyngbyastatin 8 (**1**)

Colorless amorphous solid; [α]^20^_D_ −4 (*c* 0.02, MeOH); UV (MeOH) *λ*_max_ (log *ɛ*) 217 (3.80), 274 (3.12); IR (film) *ν*_max_ 3570–3000 (br), 3050, 2924, 2124 (br), 1647, 1545, 1438, 1411, 1319, 1203, 1139, 1019, 952 cm^−1^; ^1^H-NMR, edited HSQC, HMBC and ROESY, see Table 1 and Table S1, Supporting Information; HRESI/APCIMS *m/z* [M + Na]^+^ 955.4524 (calcd for C_47_H_64_N_8_O_12_Na, 955.4541), [M + H – H_2_O] 915.4599 (calcd for C_47_H_63_N_8_O_11_, 915.4616).

### 3.4. Lyngbyastatin 9 (**2**)

Colorless amorphous solid; [α]^20^_D_ −16 (*c* 0.02, MeOH); UV (MeOH) *λ*_max_ (log *ɛ*) 212 (3.80), 272 (3.12); IR (film) *ν*_max_ 3700–3000 (br), 2956, 2923, 2854, 2361, 1729, 1643, 1540, 1451, 1409, 1380, 1257, 1205, 1073, 1025, 802, 752, 702 cm^−1^; ^1^H-NMR, edited HSQC, HMBC and ROESY, see Table 1 and Table S2, Supporting Information; HRESI/APCIMS *m/z* [M + Na]^+^ 983.4823 (calcd for C_49_H_68_N_8_O_12_Na, 983.4854), [M + H – H_2_O] 943.4898 (calcd for C_49_H_67_N_8_O_11_, 943.4929).

### 3.5. Lyngbyastatin 10 (**3**)

Colorless amorphous solid [24]; [α]^20^_D_ −36 (*c* 0.009, MeOH); UV (MeOH) *λ*_max_ (log *ɛ*) 204 (4.25), 230(sh) (3.80), 280 (3.12); ^1^H-NMR, COSY, edited HSQC, and ROESY, see Table 1 and Table S3, Supporting Information; HRESI/APCIMS *m/z* [M + Na]^+^ 1061.3951, 1063.3944 (ratio 1:1.2, calcd for C_49_H_67_N_8_O_12_^79^BrNa, 1061.3959; C_49_H_67_N_8_O_12_^81^BrNa, 1063.3939), [M + H – H_2_O] 1021.4028, 1023.4033 (ratio 1:1.2, calcd for C_49_H_66_N_8_O_11_^79^Br, 1021.4034; C_49_H_66_N_8_O_11_^81^Br, 1023.4014).

### 3.6. ESIMS Fragmentation

Solutions of compounds **1**–**3** were directly injected into the mass spectrometer by syringe driver. Spectra were collected in positive ion mode, using Enhanced Product Ion (EPI) scans. [M + Na]^+^ peaks were fragmented (*m*/*z* 955.2 for **1**, 983.5 for **2** and 1061.6/1063.4 for **3**), by ramping CE through the maximum possible range. Source parameters used were as follows: CUR 10, CAD High, IS 5500, TEM 0, GS1 10, GS2 10. Compound dependent parameters used for **1** were as follows: DP 321, EP 10, CEP 40; for **2**: DP 119, EP 11, CEP 37; and for **3**: DP 112, EP 10, CEP 40. For some of the lower molecular weight fragment ions, conventional MS^2^ scans were used to fragment the same peaks. Again, CE was ramped during the scans. Source parameters used were as follows: CUR 10, IS 5500, TEM 200, GS1 10, GS2 20. Compound dependent parameters used for **1** were as follows: DP 150, EP 4, CEP 40; for **2**: DP 140, EP 12, CEP 40; and for **3**: DP 150, EP 12, CEP 40.

### 3.7. Marfey’s Analysis

Samples (~100 μg) of compounds **1** and **2** were treated with 6 N HCl at 110 °C for 24 h. The hydrolysates were evaporated to dryness and dissolved in H_2_O (100 μL). To this 1 M NaHCO_3_ (50 μL) and a 1% ^w^/_v_ solution of 1-fluoro-2,4-dinitro-5-l-leucinamide (l-FDLA) in acetone was added, and the mixture was heated at 80 °C for 3 min. The reaction mixture was then cooled, acidified with 2 N HCl (100 μL), dried, and dissolved in H_2_O–MeCN (1:1). Aliquots were subjected to reversed-phase HPLC (Alltech Alltima HP C18 HL 5 μm, 250 × 4.6 mm, 1.0 mL/min, PDA detection) using a linear gradient of MeCN in 0.1% ^v^/_v_ aqueous TFA (30–70% MeCN over 50 min). The retention times (*t*_R_, min) of the derivatized amino acids in the corresponding hydrolysates of compounds **1** and **2** matched those of l-Thr (14.0), l-Ala (19.6), l-Val (23.9), l-Phe (28.8) and *N*-Me-l-Tyr (40.9). Retention times (*t*_R_, min) of authentic standards were as follows: l-Thr (14.0), d-Thr (19.3), l-*allo*-Thr (15.1), d-*allo*-Thr (17.0), l-Ala (19.6), d-Ala (24.0), l-Val (23.9), d-Val (32.7), l-Phe (28.8), d-Phe (35.6), *N*-Me-l-Tyr (40.9), and *N*-Me-d-Tyr (42.3) [25].

CrO_3_ oxidations of **1** and **2** followed by acid hydrolysis were carried out as previously described [6]. The resulting hydrolysates were derivatized with l-FDLA and aliquots subjected to reversed-phase HPLC as above. When compared to the above results, HPLC profiles for derivatives resulting from compounds **1** and **2** showed one new peak corresponding to l-Glu (*t*_R_ 16.5 min), and the peak corresponding to l-Phe (*t*_R_ 28.8 min) showed increased intensity. The retention times of authentic standards for l-Glu and d-Glu were 16.5 and 17.7 min, respectively.

A sample (50 μg) of **3** was hydrolyzed then derivatized with l-FDLA in the same manner as **1** and **2**. Aliquots were subjected to reversed-phase HPLC (Alltech Alltima HP C18 HL 5 μm, 250 × 4.6 mm, 0.5 mL/min, MS detection in negative ion mode) using a linear MeOH–H_2_O gradient (both containing 0.1% HCOOH, 40–100% MeOH over 50 min). l-Thr, *N*-Me-3’-Br-l-Tyr, l-Ala, l-Val and l-Phe eluted at *t*_R_ 33.7, 37.5, 38.5, 39.8, and 41.8 min, respectively. Using the same conditions, the presence of l-Thr, l-Ala, l-Val and l-Phe was confirmed in **1** and **2**. The retention times (*t*_R_, min; Multiple Reaction Monitoring (MRM) ion pair, parent→product) of authentic standards were as follows: l-Thr (33.7; 412→306), l-*allo*-Thr (36.0; 412→306), d-*allo*-Thr (37.9; 412→306), d-Thr (40.0; 412→306), *N*-Me-3’-Br-l-Tyr (37.5; 568→475 and 566→473), *N*-Me-3’-Br-d-Tyr (39.5; 568→475 and 566→473) [26], l-Ala (38.5; 382→320), d-Ala (43.8; 382→320), l-Val (39.8; 410→348), d-Val (47.9; 410→348), l-Phe (41.8; 458→396), and d-Phe (49.5; 458→396). MS parameters used for detection of the majority of standards were as follows: DP −57, EP −8, CE −18, CXP −17, CUR 50, CAD High, IS −4500, TEM 750, GS1 40, GS2 50. For detection of *N*-Me-3’-Br-Tyr, MS parameters used were as follows: DP −90, EP −8, CE −40, CXP −21, CUR 50, CAD High, IS −4500, TEM 750, GS1 40, GS2 50.

### 3.8. Elastase Assays

A stock solution of porcine pancreatic elastase (Elastase-high purity; EPC, EC134) was prepared at a concentration of 75 μg/mL in Tris-HCl (pH 8.0). Aliquots of test compounds (1 μL, DMSO) were preincubated with 79 μL Tris-HCl (pH 8.0) and 5 μL elastase stock for 15 min at room temperature in a microtiter plate. After this time, 15 μL substrate solution was added (2 mM *N*-succinyl-Ala-Ala-Ala-*p*-nitroanilide in Tris-HCl, pH 8.0) to each well, and the reaction was followed by measuring the absorbance at 405 nm every 30 s. Enzyme activity was determined by calculating the initial slope of each progress curve, expressed as a percentage of the slope of the uninhibited reaction. Lyngbyastatin 7 [7] was used as a positive control for inhibition, and the assay was carried out in triplicate.

## Figures and Tables

**Table 1.** NMR Spectral Data for Lyngbyastatin 8–10 (**1**–**3**) in DMSO-*d*_6_ at 600 MHz (^1^H).

**C/H No.**	**Lyngbyastatin 8 (1)**		**Lyngbyastatin 9 (2)**		**Lyngbyastatin 10 (3)**	
	**δ_H_, mult. (*J*in Hz)**	**δ_C_, mult.*a***	**δ_H_, mult. (*J*in Hz)**	**δ_C_, mult.*a***	**δ_H_, mult. (*J*in Hz)**	**δ_C_, mult.*a***
1		*c*		*c*		*c*
2	4.64, br	55.9, d	4.62, m	55.9, d	4.60, m	56.2, d
3	2.05, m	30.4, d	2.05, m	30.3, d	2.03, m	30.4, d
4	0.86, d (6.6)	19.0, q	0.85*d*	18.9, q	0.86, d (7.0)	19.0, q
5	0.74, d (6.6)	17.2, q	0.74, d (6.6)	17.1, q	0.74, d (7.0)	17.2, q
NH	7.50, br d (6.3)		7.52, br d (6.8)		7.52, br d (7.3)	
6		*c*		*c*		*c*
7	4.87, dd (11.8, 1.9)	60.6, d	4.87, dd (12.0, 0)	60.5, d	4.86, dd (12.0, 2.4)	60.6, d
8a	3.08, dd (−13.4, 1.9)	32.5, t	3.08, dd (−13.0, 0)	32.4, t	3.08, dd (−13.6, 2.4)	32.2, t
8b	2.69, dd (−13.4, 11.8)		2.69, dd (−13.0, 12.2)		2.72, dd (−13.6, 12..0)	
9		127.3, s		127.2, s		*c*
10	6.97, d (8.0)	130.3, d	6.97, d (7.6)	130.2, d	7.25, s	133.4, d
11	6.76, d (8.0)	115.2, d	6.76, d (7.6)	115.1, d		*c*
12		156.2, s		156.0, s		*c*
13	6.76, d (8.0)	115.2, d	6.76, d (7.6)	115.1, d	6.93, d (8.2)	116.5, d
14	6.97, d (8.0)	130.3, d	6.97, d (7.6)	130.2, d	6.97, d (8.2)	130.0, d
15	2.74, s	30.2, q	2.74, s	30.1, q	2.75, s	30.3, q
OH	9.38, s		9.41, s		8.48, s	
16		170.6, s		170.3, s		*c*
17	4.71, dd (12.1, 3.1)	50.0, d	4.71, dd (12.1, 3.0)	49.9, d	4.70, dd (11.7, 3.2)	50.2, d
18a	2.86, dd (−12.8, 12.1)	35.0, t	2.85, dd (−12.8, 12.1)	35.0, t	2.88, dd (−13.7, 11.7)	35.2, t
18b	1.80, dd (−12.8, 3.1)		1.80, dd (−12.8, 3.0)		1.87, dd (−13.7, 3.2)	
19		136.8, s		136.5, s		*c*
20/24	6.82, d (7.2)	129.3, d	6.82, d (7.3)	129.2, d	6.78, d (7.4)	129.5, d
21/23	7.18, m	127.7, d	7.18, m	127.6, d	7.16, dd (7.4, 7.3)	128.0, d
22	7.14, m	126.1, d	7.14, m	126.0, d	7.13, t (7.3)	126.4, d
25		*c*		168.5, s		*c*
26	3.77, ddd (12.4, 9.2, 2.4)	47.9, d	3.77, ddd (14.1, 10.6, 2.3)	47.9, d	3.78, ddd (11.6, 8.8, 2.2)	48.2, d
27a	2.40, dddd (−12.4, 12.4, 11.7, 2.7)	21.7, t	2.40, dddd (14.1, −12.4, 11.3, 4.4)	21.6, t	2.40, m	21.8, t
27b	1.56, m		1.56, m		1.56, m	
28a	1.70, br d (11.7)	29.1, t	1.71, br d (11.3)	29.0, t	1.71, br d (12.4)	29.2, t
28b	1.56, m		1.55, m		1.57, m	
29	5.06, s	73.5, d	5.06, s	73.4, s	5.07, s	73.7, d
NH	7.21, d (9.2)		7.21, br		7.21, br	
OH	6.10, s		6.11, br		3.15, s*e*	
30		*c*		162.7, s		*c*
31		130.1, s		129.7, s		*c*
32	6.49, q (6.8)	131.6, d	6.49, q (6.8)	131.5, d	6.50, q	132.1, d
33	1.47, d (6.8)	12.9, q	1.47, d (6.8)	12.8, q	1.47, d	12.9, q
NH	6.56, s		6.58, s			
34		*c*		*c*		*c*
35	4.62, m	55.1, d	4.63, m	55.0, d	4.61, m	55.7, d
36	5.53, br	71.4, d	5.53, br	71.4, d	5.50, br	*c*
37	1.21, d (6.2)	17.6, q	1.21, d (6.2)	17.6, q	1.21, d (6.7)	17.8, q
NH	7.91, br		7.92, br		7.92, br	
38		*c*		171.7, s		*c*
39	4.36, m	57.0, d	4.37, m	56.8, d	4.36, dd (8.8, 6.1)	57.1, d
40	2.05, m	30.4, d	2.05, m	30.3, d	2.03, m	30.4, d
41	0.85, d (6.2)	19.0, q	0.84*d*	13.3, q	0.85, d (6.1)	19.0, q
42	0.80, d (6.4)	17.5, q	0.80, d (6.5)	17.4, q	0.80, d (6.6)	17.5, q
NH	7.77, br		7.69, br		7.68, br	
43		172.5, s		172.3, s		*c*
44	4.33, dq (7.4, 6.7)	47.8, d	4.34, dq (7.2, 6.8)	47.7, d	4.32, dq (7.6, 7.0)	47.9, d
45	1.17, d (6.7)	17.8, q	1.18, d (6.8)	17.6, q	1.18, d (7.0)	17.8, q
NH	8.08, d (7.4)		8.04, d (7.2)		8.01, d (7.6)	
46		169.0, s		171.8, s		*c*
47	1.82, s	22.2, q	2.07, m (2H)	36.7, t	2.07, m (2H)	36.8, t
48			1.48, m (2H)	18.3, t	1.48, m (2H)	18.5, t
49			0.83*d*	13.3, q	0.83, t (7.3)	13.4, q

^a^ Deduced from edited HSQC.

^b^ Protons showing HMBC correlations to the indicated carbon.

^c^ Could not be detected due to lack of HMBC correlation.

^d^ The multiplicity of these signals could not be deduced due to signal overlap.

^e^ Assigned by default as no COSY correlations were observed. Chemical shift is different to **1** and **2** probably because of different sample concentrations (data for **1** and **2** were acquired with a 1-mm probe and therefore they were much more concentrated).

## Glossary of Terms

Abu: 2-amino-2-butenoic acid;
Ahp: 3-amino-6-hydroxy-2-piperidone;
Amp: 3-amino-6-methoxy-2-piperidone;
Ba: butanoic acid;
CAD: collisionally activated decomposition;
CE: collision energy;
CEP: collision cell entrance potential;
CUR: curtain gas;
DP: declustering potential;
EP: entrance potential;
EPI: enhanced product ion;
FDLA: 1-fluoro-2,4-dinitro-5-leucinamide;
Ga: glyceric acid;
Gas: glyceric acid sulfate;
GS1: gas 1;
GS2: gas 2;
Ha: hexanoic acid;
HRESI/APCIMS: high resolution electrospray ionization/atmospheric pressure chemical ionization mass spectrometry (dual probe);
Htyr: homotyrosine;
IS: ionspray voltage;
TEM: temperature.
